# Muscle-on-a-chip devices: a new era for *in vitro* modelling of muscular dystrophies

**DOI:** 10.1242/dmm.050107

**Published:** 2023-06-23

**Authors:** Juan M. Fernández-Costa, Ainoa Tejedera-Vilafranca, Xiomara Fernández-Garibay, Javier Ramón-Azcón

**Affiliations:** ^1^ Institute for Bioengineering of Catalonia (IBEC), The Barcelona Institute of Science and Technology (BIST), Baldiri Reixac 10-12, 08028 Barcelona, Spain; ^2^ Institució Catalana de Reserca i Estudis Avançats (ICREA), Passeig de Lluís Companys, 23, 08010 Barcelona, Spain

## Abstract

Muscular dystrophies are a heterogeneous group of highly debilitating diseases that result in muscle atrophy and weakness. The lack of suitable cellular and animal models that reproduce specific aspects of their pathophysiology is one of the reasons why there are no curative treatments for these disorders. This highlights a considerable gap between current laboratory models and clinical practice. We strongly believe that organs-on-chip could help to fill this gap. Organs-on-chip, and in particular muscles-on-chip, are microfluidic devices that integrate functional skeletal muscle tissues. Biosensors in these systems allow monitoring of muscle homeostasis or drug responses *in situ*. This Perspective outlines the potential of organs-on-chip as advanced models for muscular dystrophies, as well as the current challenges and future opportunities for this technology.

## Teaser

**Summary:** Organ-on-a-chip devices, which combine engineered 3D skeletal muscle tissues and integrated biosensors, hold great promise as a tool for studying muscular dystrophies and developing new therapies for these conditions.

## Introduction

Under the name of muscular dystrophies, we find a group of heterogeneous genetic diseases that differ in the pattern of inheritance, age of onset, progression, or even in the type of muscle affected, but all have muscle degeneration as a common feature (Mercuri et al., 2019). To date, more than 70 genetic muscular dystrophies have been described, which can be grouped into nine categories: Duchenne, Becker, myotonic, limb-girdle, facioscapulohumeral, congenital, oculopharyngeal, distal and Emery-Dreifuss (Benarroch et al., 2019; Carter et al., 2018). Individually, these disorders could be considered rare diseases. For example, the most prevalent muscular dystrophy is myotonic dystrophy type 1, with a recently redefined prevalence of 1 in every 2100 births (Johnson et al., 2021). However, taken together, they have a significant impact on society, globally. Most of these diseases have no curative treatment, reducing the options for patients to palliative drug treatments and other palliative approaches, such as physiotherapy. Finding curative therapies for these diseases has become an imperative unmet clinical need. Therefore, accelerating drug development for these diseases is crucial, and simple, high-throughput and accurate approaches for testing drugs *in vitro* are needed for this to be achieved.

Drug development for muscular dystrophies has exploded in the past decade due to the increased understanding of muscular dystrophy pathophysiology and advances in gene therapies and drug technologies (Roy and Griggs, 2021). Therefore, pharmaceutical companies have numerous drugs for these diseases in their pipeline. However, these molecules must demonstrate their efficacy and reduced toxicity in humans to become approved treatments. Preclinical studies with *in vitro* and animal models provide extensive preliminary data on a candidate drug's efficacy, toxicity, pharmacokinetics and safety. Nevertheless, despite the effort and money invested in the preclinical phases, 90% of the candidate drugs in clinical trials fail (Dowden and Munro, 2019). The drug's high toxicity or low efficacy in humans is the underlying reason for this high failure rate. The vast difference between data from preclinical studies and clinical trials indicates that current preclinical models are insufficient. Therefore, advanced models are needed to improve the success rate of drug development. In addition, the intrinsic heterogeneity of muscular dystrophies suggests that each patient would respond to treatments differently. Hence, these advanced models should include patient-derived cells to test personalized therapies.

**Figure:**
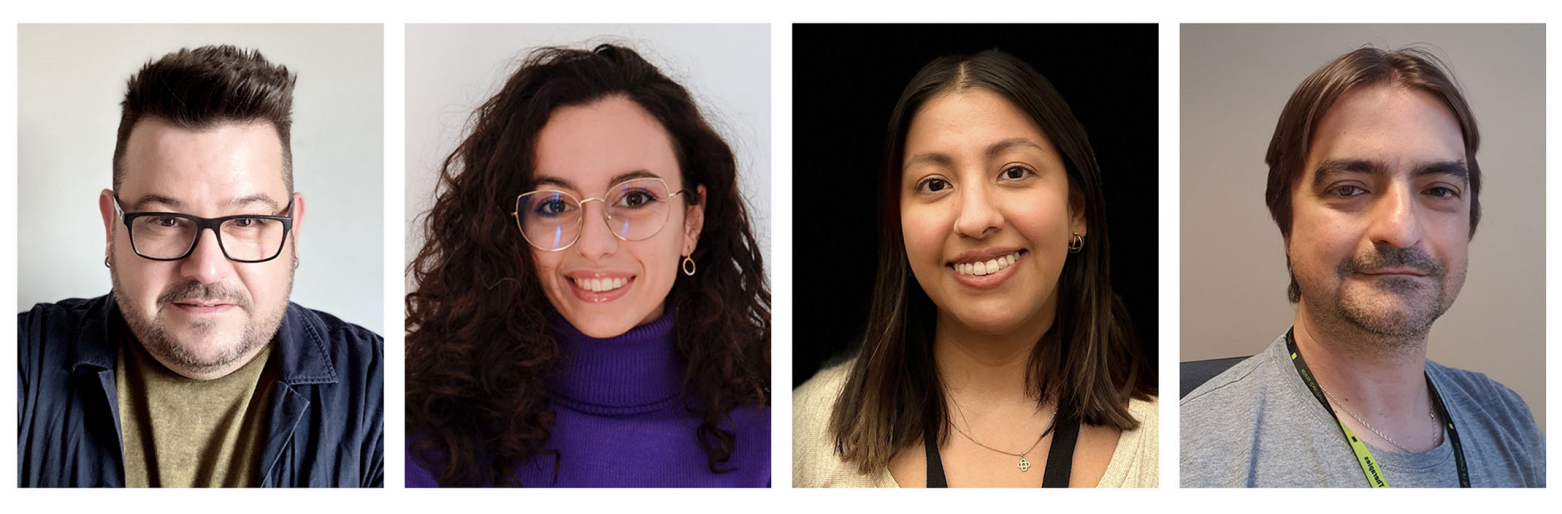
Juan M. Fernández-Costa, Ainoa Tejedera-Vilafranca, Xiomara Fernández-Garibay and Javier Ramón-Azcón (left to right)

## *In vitro* models for muscular dystrophies fail to reproduce relevant phenotypes

Owing to the complex structure of skeletal muscle, traditional cellular models do not fully recapitulate their features *in vitro*. Skeletal muscle function is directly correlated with the aligned structure of myofibres (see Glossary, Box 1). For this reason, tissue engineering aims to reproduce this aligned structure, providing geometric cues to the cellular cultures (Fernández-Costa et al., 2021). Engineered skeletal muscle models provide new physiological phenotypes that could be used for drug screening studies, such as functional phenotypes related to contractile force or structural phenotypes replicating myotube (Box 1) diameter (Ebrahimi et al., 2021; Fernández-Garibay et al., 2021; Maffioletti et al., 2018; Oliver et al., 2022; Vesga-Castro et al., 2022). Although these new *in vitro* models provide more relevant phenotypes for drug screening, we must go a step further and develop models for muscular dystrophies that not only integrate functional tissues but also monitor their homeostasis.

**Box 1. Glossary**

**Bioreactor:** a device that is used to support and control the growth of cells, tissues or microorganisms.

**Fibroadipogenic precursors (FAPs):** a group of muscle progenitor cells that contribute to muscle regeneration and maintenance.

**Geometrical confinement:** cell seeding on top of surfaces that are micropatterned (Hosseini et al., 2012; Nikkhah et al., 2012) or encapsulation of the cells in filaments using micromolding (Costantini et al., 2017; de Chiara et al., 2022; Lopez-Munõz et al., 2021; Ortega et al., 2019).

**Myoblasts:** muscle precursor cells that have the ability to fuse and differentiate to myotubes.

**Myofibres:** basic units of muscle tissue, responsible for muscle contraction.

**Myotube:** an elongated, multinucleated cell that forms as a result of the fusion of myoblasts and constitutes the precursor of myofibres.

**Optical/magnetic detection of contraction by measurement of cantilever displacement:** magnetic sensing platforms, in which tissues are fabricated around one rigid and one flexible post, with an embedded magnet in each flexible post. The system is built so that the magnetic field at the sensors changes in response to tissue contraction, resulting in an output voltage change that is recorded in a software suite and translated into post-deflection and exerted tissue force.

**Plasmonic biosensor:** a type of biosensor that uses optical properties of the materials to detect and measure molecules, such as proteins.

**Post-displacement measurement:** a technique used to analyse the force exerted by a bioengineered skeletal muscle tissue by measuring the deflection of a post to which the tissue is attached, following electric pulse stimulation.

**Sarcomere:** the basic functional unit of a muscle fibre. It is composed of a repeating pattern of actin and myosin filaments, which slide past each other during muscle contraction, causing the muscle to shorten.

**Shear stress**: a type of stress that occurs when two parallel layers of a material move in opposite directions. Shear stress applied to the myoblasts using extrusion-based techniques, such as 3D bioprinting or electrospinning, can pre-align them for its differentiation in aligned myotubes (Alheib et al., 2021; García-Lizarribar et al., 2018; Kim and Kim, 2020).

**Uniaxial tension:** use of tendon-like anchoring sites from which the biomaterial containing the cells is hung. This promotes the myotubes to form and fuse in an aligned direction along the axis of the anchoring points.

## What can organs-on-chip offer to muscular dystrophies?

**Fig. 1.**
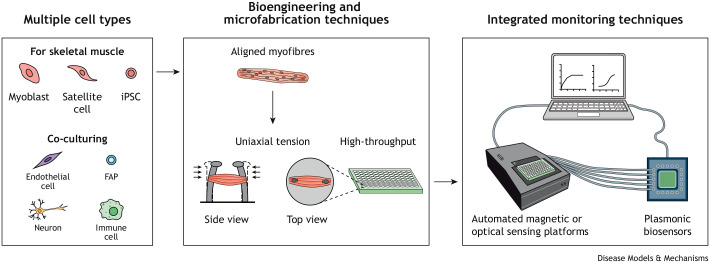
**Muscle-on-a-chip technology.** Advanced muscle-on-a-chip devices should incorporate different cell types, including cells derived from patients, and use bioengineering techniques to recapitulate muscle function and miniaturize the tissue for high-throughput capacity. Integrating these systems with non-invasive and real-time monitoring techniques, such as automated magnetic or optical sensing platforms, or plasmonic biosensors for the detection of relevant biomarkers, will further enhance their potential in preclinical research. FAP, fibroadipogenic precursor; iPSC, induced pluripotent stem cell.

In 2010, the term organ-on-a-chip (OOC) was invented by Donald Ingber and his team, who developed a microfluidic chip integrating organ-level physiological functions of the human lung (Huh et al., 2010). OOC devices, in general, are microphysiological systems that recapitulate human organs and their (patho-)physiological responses to specific cues or compounds by integrating complex cellular cultures. Muscle-on-a-chip (MOC) devices consist of a bioreactor (Box 1) that reproduces the microphysiological environment of the muscle and allows its electrical stimulation to induce muscle contraction (Agrawal et al., 2017; Ortega et al., 2019; Osaki et al., 2020) (Fig. 1). This bioreactor must integrate functional three-dimensional (3D) skeletal muscle tissues that respond to electrical stimulation. Thus, 3D muscle cultures must be developed in biomaterial scaffolds building highly organized cellular microarchitecture. The MOC bioreactors are connected by a microfluidic system that allows the flow of tissue culture medium to be controlled, enabling the monitored administration of drugs.

We consider the development of MOCs to contribute to muscular dystrophy studies in three main ways: by deepening the understanding of the basis of the disease pathology, by accelerating the development of drugs with more relevant outcomes, and by supporting the study of personalized research through the introduction of cells derived from individual patients. MOC devices could be the solution for the lack of good preclinical models for muscular dystrophy. Moreover, although they cannot completely replace the use of animals in drug development, the fact that we can test drugs in MOCs that harbour cells from patients and mimic a given physiological function will undoubtedly contribute to reducing the number of animals in research.

“MOC devices could be the solution for the lack of good preclinical models for muscular dystrophy.”

## Introducing engineered skeletal muscle tissues in OOCs

To develop relevant MOC devices, it is of high importance that the skeletal muscle tissues that are incorporated reproduce the pathophysiological events in which we are interested. The MOC must recapitulate as precisely as possible the main characteristics and functions of native skeletal muscle, especially the ones that are involved in the specific muscular dystrophy of study. However, the complexity of skeletal muscle tissue challenges this achievement. Skeletal muscle includes a wide range of cellular types that interact with each other in muscle function, regeneration and disease (Bentzinger et al., 2013). These cellular types include immune cells, fibroadipogenic precursors (FAPs; Box 1), blood vessel-associated cells, and committed and differentiated myoblasts (Box 1), as well as a muscle stem cell niche. Nevertheless, the main cell type present in skeletal muscle and the one that supports the main functions of the organ, such as muscle contraction, structural support or metabolic regulation, is the myotube (Frontera and Ochala, 2015). Thus, because of their relevance, skeletal muscle cell culture has typically focused on myotubes.

The complex organization of skeletal muscle tissue, based on aligned and multinucleated myofibres that contain the contractile sarcomere (Box 1) units, directly influences its functionality (Mukund and Subramaniam, 2020). Traditional two-dimensional (2D) cell culture fails to achieve aligned differentiation of myoblast culture into myotubes. Therefore, bioengineering techniques have focused on improving myotube differentiation status. Guiding the alignment of myogenic precursors is important to recapitulate the formation of organized myotubes and achieve complex 3D skeletal muscle models. Bioengineering strategies are based on the incorporation of different topographical and mechanical cues that influence myogenic precursors to differentiate into aligned and organized myotubes. Geometrical confinement (Box 1) and shear stress (Box 1) are some of the first strategies that achieved aligned, organized, multinucleated myotubes. These bioengineering strategies can develop diseased skeletal muscle models with more complexity, and they could help identify new disease phenotypes (Fernández-Garibay et al., 2021).

However, these methodologies failed to achieve the proper differentiation status of the myotubes to allow contractility. In recent years, uniaxial tension (Box 1) appeared as an alternative strategy. The 3D skeletal muscle tissues generated are differentiated enough to achieve functionality, and they respond to electrical stimuli by contracting (Abdel-Raouf et al., 2021; Bakooshli et al., 2019; Fernández-Garibay et al., 2022; Madden et al., 2015; Mestre et al., 2019; Rao et al., 2018). Using this strategy, researchers have been able to create skeletal muscle models using several cell types, such as skeletal muscle precursors, immortalized and primary myoblasts, or induced pluripotent stem cells, which include cells from patients with various muscular diseases (Ebrahimi et al., 2021; Maffioletti et al., 2018; Oliver et al., 2022; Shahriyari et al., 2022). This is a significant advance in the skeletal muscle bioengineering field and has implications for bioengineering other contractile tissues, such as cardiac muscle. Moreover, it opens an exciting door to the development of complex OOCs with potential implications for research into various different muscular dystrophies. However, an additional challenge is to adapt the current models to fit medium- to high-throughput screening applications. Although scaling down the physical size of the platform is possible with current microfabrication technologies, tissue miniaturization must be achieved without compromising the bioengineered skeletal muscle tissue architecture and its components.

“The use of OOC devices can improve the predictivity of preclinical studies by better mimicking human physiology, reducing the use of animals and ultimately improving the efficiency of drug development.”

## Integrating monitoring tools in OOCs

For the continuous improvement of cell monitoring and OOC systems, there is a necessity to integrate functional and real-time monitoring tools (Mughal et al., 2022; Oomen et al., 2016; Rogal et al., 2017). Current OOC cell culture analysis is mainly based on optical measurement techniques using fluorescence microscopy (Vinci et al., 2012). However, fluorescent labelling is both a qualitative method and a terminal assay, and all the real-time metabolic behaviour is lost. OOC integrated with new sensing technology allows easier intracellular and extracellular measurements across different tissues. For these reasons, *in vitro* devices capable of studying the crosstalk between diverse cell populations allow for further understanding of complex systems and could contribute to the integration and study of multiorgan devices. Owing to the different metabolic processes of biomimetic tissues, this monitorization can be performed inside and outside the cell culture area using measurement systems connected with the OOC without interfering with their function. There are a few examples of OOCs with 3D functional tissues and in-built sensors, such as those for oxygen (Wu et al., 2010; Yip et al., 2014), pH (Mousavi Shaegh et al., 2016), glucose (Obregón et al., 2013) and lactate (Weltin et al., 2014). However, most of these systems cannot be widely applied in microfluidic systems, or do not work in real time, and they do not provide biomarker information for specific muscular dystrophy pathology.

Some examples can be found that have fabricated modular platforms to detect specific biomarkers, such as insulin, TNF-α (TNF) or IL6 (Hernández-Albors et al., 2019; Ortega et al., 2019), from complex biological *in vitro* media. Unfortunately, biosensors based on oxidation–reduction reactions, fluorescence or enzymatic activity need incubation and washing steps, thus not providing real-time measurements and limiting the value of the data acquired. In this scenario, plasmonic biosensors (Box 1) have become widespread label-free sensing tools in different fields, including medical diagnosis (Homola, 2003), and have many advantageous properties, including the possibility of performing real-time, *in situ* analyses without additional incubation or washing steps. Considering such potential improvements in biosensing performance and integration, plasmonic biosensors based on nanostructured materials (Homola, 2008) can surpass conventional plasmonic sensors' challenges. Recently, some works have been published in which OOCs have been integrated with plasmonic biosensors. This technology has been successfully applied to liver (Lopez-Muñoz et al., 2020), muscle (Lopez-Munõz et al., 2021) and pancreas (Ortega et al., 2021) engineered tissue. Although the working range of these sensors is very wide, and saturation effects are not expected, longitudinal monitoring has not yet been tested to date.

Ultimately, the expectations in this field are for a new generation of optical label-free biosensors. These new biosensors will be made with the potential to measure concentrations below pg ml^−1^ of nanovesicles, such as exosomes, and work in a kinetic configuration allowing real-time measurements of protein biomarkers, such as myokines or muscle damage protein markers, in a multiplexed way. We suggest that integration of these plasmonic biosensors in MOC devices is a very powerful solution for monitoring muscles responses by analysing the muscle secretome in real time.

Furthermore, readouts of muscle functionality will need to be automated to be compatible with high-throughput assays. Currently, the most widely used methods for functionality readouts involve invasive force transducers (Khodabukus et al., 2019; Madden et al., 2015; Rao et al., 2018; Steele-Stallard et al., 2018) or video processing and analysis of individual samples for post-displacement measurement (Box 1) (Bakooshli et al., 2019; Christensen et al., 2020; Fernández-Costa et al., 2022; Fernández-Garibay et al., 2022; Vandenburgh et al., 2008). Although measuring post displacement is a desirable strategy due to its non-invasive nature, the analysis is extremely time consuming and requires trained scientists to obtain reproducible measurements. In this regard, we are confident that non-invasive but automated methods, such as optical or magnetic detection of contraction by measurement of cantilever displacement (Box 1), will be essential in future skeletal MOC devices (Smith et al., 2022). Remarkably, these analyses require no expertise from the operator.

“Human-on-a-chip would provide a more comprehensive understanding of how drugs can affect the body and help to identify potential side effects before they occur in human trials.”

## Future directions and challenges for OOCs to study muscular dystrophies

The advances in human skeletal muscle tissue engineering and functional 3D *in vitro* models can improve our knowledge of muscle pathologies. However, their integration with OOCs remains limited, and only a few proof-of-concept works have been published to date (Agrawal et al., 2017; Fernández-Costa et al., 2022; Mondrinos et al., 2021; Ortega et al., 2019). The overall goal will be to increase the tissue complexity while decreasing the technological complexity so OOCs become standard laboratory techniques for the study of muscle diseases and for preclinical research. Increasing the complexity of tissues involves introducing co-cultures of other cell types besides myoblasts, such as FAPs, immune system cells or endothelial cells. Owing to the impact of inflammation and fibrosis in muscular dystrophy pathogenesis, we suggest prioritizing co-cultures with FAPs and immune system cells.

The use of OOC devices can improve the predictivity of preclinical studies by better mimicking human physiology, reducing the use of animals and ultimately improving the efficiency of drug development. OOCs can be used to create patient-specific models by integrating patient-derived cells, to identify the best treatment options for individual patients. In our view, the use of OOCs as platforms for personalized drug testing using patient-specific cells is undoubtedly one of the most important and promising uses of these devices for muscular dystrophies. Owing to the intrinsic heterogeneity of these diseases, both phenotypes and drug response can vary greatly from patient to patient, so these personalized platforms may be the solution to finding the treatment that works best for each patient.

In addition, OOC devices can be used to study the interactions between different organs and how drugs can affect these systemic interactions. Multi-OOCs integrate different 3D tissues connected by microfluidic channels and allow the study of communication between these organs. In this way, multi-OOCs mimic complex physiological interactions between different organs, such as blood flow, oxygen and nutrient supply, and communication via hormones and other signalling molecules (Novak et al., 2020; Ronaldson-Bouchard et al., 2022). In the context of muscular dystrophy modelling, it may be worthwhile to consider incorporating cardiac tissue along with skeletal muscle in a multi-OOC system, given that some forms of these diseases affect both types of muscle tissue. Recently, this multi-OOC has been developed to study systemic toxic effects of potential drugs *in vitro* (McAleer et al., 2019). The ultimate objective of multi-OOC is the human-on-a-chip, a device that contains all the organs in the human body allowing the study of human physiology *in vitro*. Human-on-a-chip would provide a more comprehensive understanding of how drugs can affect the body and help to identify potential side effects before they occur in human trials.

Finally, we want to highlight the need for standardization to bring OOC technology to the pharmaceutical industry. Currently every laboratory in the world working on OOC development has its own manufacturing protocols. Standardization of fabrication and quality control protocols for OOC production are indispensable for their large-scale use in laboratories.

In our opinion, the future of the study of muscular dystrophies lies with OOC technology. Although there are still challenges in making them a widely applied standard research technique, they are already contributing to the understanding of pathogenesis and the search for drugs for muscular dystrophies.
